# *Wolbachia* strain diversity in a complex group of sympatric cryptic parasitoid wasp species

**DOI:** 10.1186/s12866-024-03470-7

**Published:** 2024-09-02

**Authors:** Federica Valerio, Cornelia Martel, Constanti Stefanescu, Saskya van Nouhuys, Maaria Kankare, Anne Duplouy

**Affiliations:** 1 Organismal and Evolutionary Biology Research Programme, University of Helsinki, Helsinki, Finland; 2 Department of Biology, Lund University, Lund, Sweden; 3 Natural Sciences Museum of Granollers, Granollers, Spain; 4 Centre for Ecological Sciences, Indian Institute of Science, Bangalore, India; 5 Department of Biological and Environmental Science, University of Jyväskylä, Jyväskylä, Finland

**Keywords:** Microbial symbiosis, Hymenoptera, Melitaeini butterflies, *Cotesia*, Cytoplasmic incompatibility

## Abstract

**Background:**

Maternally-inherited symbionts can induce pre-mating and/or post-mating reproductive isolation between sympatric host lineages, and speciation, by modifying host reproductive phenotypes. The large parasitoid wasp genus *Cotesia* (Braconidae) includes a diversity of cryptic species, each specialized in parasitizing one to few related Lepidoptera host species. Here, we characterized the infection status of an assemblage of 21 *Cotesia* species from 15 countries by several microbial symbionts, as a first step toward investigating whether symbionts may provide a barrier to gene flow between these parasitoid host lineages.

**Results:**

The symbiotic microbes *Arsenophonus*, *Cardinium*, Microsporidium and *Spiroplasma* were not detected in the *Cotesia* wasps. However, the endosymbiotic bacterium *Wolbachia* was present in at least eight *Cotesia* species, and hence we concentrated on it upon screening additional DNA extracts and SRAs from NCBI. Some of the closely related *Cotesia* species carry similar *Wolbachia* strains, but most *Wolbachia* strains showed patterns of horizontal transfer between phylogenetically distant host lineages.

**Conclusions:**

The lack of co-phylogenetic signal between *Wolbachia* and *Cotesia* suggests that the symbiont and hosts have not coevolved to an extent that would drive species divergence between the *Cotesia* host lineages. However, as the most common facultative symbiont of *Cotesia* species, *Wolbachia* may still function as a key-player in the biology of the parasitoid wasps. Its precise role in the evolution of this complex clade of cryptic species remains to be experimentally investigated.

**Supplementary Information:**

The online version contains supplementary material available at 10.1186/s12866-024-03470-7.

## Background

At least 40% of all insect species worldwide are associated with endosymbiotic microbes, including *Arsenophonus*, *Cardinium*, Microsporidium, *Rickettsia*,* Spiroplasma*, and possibly the most common one: *Wolbachia* [1]. To enhance their own fitness through transmission in their host population, these microbes can manipulate their host reproduction and other life-history traits [2–4]. For example, many symbiont species can induce cytoplasmic incompatibility (CI), in which infected males are incompatible with females that are uninfected or infected with another incompatible symbiotic strain [5, 6]. Some endosymbiotic microbes can also manipulate behaviour of their host, such that infected and uninfected individuals have different mate or host preferences [7, 8, reviewed in 9]. These symbiont-induced reproductive and behavioural alterations have thus long been proposed as key drivers of host speciation and diversity [10], via post-mating isolation [11, 12], and/or pre-mating reproductive isolation between lineages of different infection status [13, 14]. For example, Shoemaker et al. [15] showed that in the *Drosophila subquinaria* species group, *Wolbachia* induces unidirectional CI, which, coupled with mate choice preferences, established a reproductive barrier between *D. recens* and *D. subquinaria*. While divergence between insect species often occurs independently of any symbiotic infection [16], the relative importance of microbial symbionts in this process is likely underestimated as the prevalence, diversity, and role of symbionts remain unknown for many insect systems.

Biogeographic studies of symbiotic diversity and prevalence, combined with phylogenetic analyses, can provide clues to the ecological and evolutionary roles of symbionts in their host species clade. For example, obligate symbionts transmitted exclusively maternally are likely to show high prevalence within their host species [17, 18], and their interactions can exhibit phylogenetic concordance between the symbiont and their host over long evolutionary periods. This pattern was, for example, observed between beneficial *Wolbachia* strains and their bedbug or nematode hosts [19, 20]. Similarly, post-mating and pre-mating reproductive isolation induced by facultative symbionts can also lead to co-divergence of the hosts and the symbionts [21], at least over short evolutionary periods required for cryptic species to diverge. However, although facultative endosymbionts are predominantly transmitted vertically from mothers to offspring, these bacteria can also be horizontally transferred between host lineages and species [22–27]. Horizontal transfer events might occur between interacting species, including between parasitoids and their prey, between prey attacked by the same parasitoid species, or between predators or parasitoids sharing the same prey [28, 29]. Transfer may also occur between herbivores sharing the same host plants [23, 24], or between hybridizing species [30]. These events allow the symbiotic strains to colonize divergent host species, which could obscure the evolution of patterns of phylogenetic concordance between host and symbiont.

Parasitoid wasps in the genus *Cotesia* (Hymenoptera: Braconidae) parasitize Lepidoptera by laying a single or multiple eggs in their host caterpillars. The parasitoid wasp larvae grow while feeding on the developing caterpillar’s haemolymph, and then pupate in conspicuous silken cocoons outside the body of the host [31]. The whole genus *Cotesia* accounts over 1000 named species worldwide, which parasitize many Lepidoptera species [32, 33]. In some cases, the *Cotesia* wasps can have dramatic effects on their host population dynamics [34]. For example, even by only infecting on average 10% of the caterpillars of *Melitaea cinxia* (Lepidoptera: Nymphalidae: Melitaeini) in the Åland Islands, Finland, *Cotesia melitaearum* has been found to cause localized decline within the larger host metapopulation [35, 36]. Furthermore, multiple *Cotesia* species can co-occur, where their host species occur together in a landscape. In North-eastern Spain, seven cryptic *Cotesia* species each use only one to two of the local eight related *Melitaea* and two *Euphydryas* (Melitaeini) butterfly species, which share some host plant species, and live in shared meadow habitats [37, 38].

To date, *Wolbachia* is, to our knowledge, the only endosymbiont that has been previously screened for, and detected from *Cotesia* species. The bacterium has been found in *C. glomerata* (Linnaeus) and *C. vestalis* (Haliday) (synonym of *C. plutellae* (Kurdjumov)) [39], and in *C. sesamiae* from Cameron and Kenya [40, 41]. Branca et al. [41] demonstrated that *Wolbachia* induces unidirectional CI in *C. sesamiae* from Sub-Saharan Africa, which is associated with host specialization, genetic structure, and biogeography. In the *C. melitaearum* clade, molecular characterizations based on small number of genes have shown that specialization and competitive interactions in local *Cotesia* are associated with the emergence of several cryptic sympatric *Cotesia* species [37, 42]. In this parasitoid wasp clade, however, the role of symbiont-induced pre-mating and/or post-mating isolation between host lineages remains to be investigated. In this study, we aimed at identifying whether common insect endosymbiotic microorganisms, including *Arsenophonus*, *Cardinium*, Microsporidium, *Spiroplasma*, and *Wolbachia*, were present in 15 *Cotesia* species and cryptic species parasitizing Melitaeini butterfly species across different geographic locations. After identifying *Wolbachia* as the only detectable symbiont in these *Cotesia* species, we characterized the diversity and phylogeny of the *Wolbachia* strains, to investigate any potential inter-species transfer of the symbionts. For this, we additionally included strains identified from six *Cotesia* species from which genomic data was publicly available on NCBI, and strains previously detected in diverse Lepidoptera species known to be attacked by *Cotesia* wasps (reviewed in [43]). Our study provides an overview of the prevalence of diverse endosymbiotic microbes in *Cotesia* wasps, as well as a more thorough description of the diversity and phylogeny of the *Wolbachia* strains detected from *Cotesia* wasps. These are the first steps towards evaluating the role such symbionts might play in the evolutionary ecology of parasitoid wasps.

## Materials and methods

### Material

We recovered 323 DNA-extracts from *Cotesia* specimens (Table S1) stored in the − 20 °C freezers at the University of Helsinki. These samples were originally collected in the early 2000’s, and used for earlier studies of the phylogeny and butterfly-host specialization of *Cotesia* species associated with checkerspot/fritillary butterflies (*Melitaea* and *Euphydryas*) [37, 42, 44]. They were previously characterized as representing at least 16 *Cotesia* species and cryptic species, and were predominantly collected from Finland (*N* = 94 from 4 species) and from Spain (*N* = 153 from 10 species). Moreover, samples from China (*N* = 8 specimens from 3 species), Estonia (*N* = 13 from 1 species), France (*N* = 24 from 6 species), Hungary (*N* = 3 from 1 species), Italy (*N* = 3 from 1 species), Russia (*N* = 11 from 1 species), Sweden (*N* = 4 from 3 species), UK (*N* = 3 from 2 species), and USA (*N* = 7 from 2 species), were also included. No tissue nor DNA material were preserved from the original butterfly hosts of these wasps.

### Molecular work on lab-stored DNA extracts

The DNA from all field collected wasps was extracted using NucleoSpin Tissue Kit (Macherey-Nagel) for the purpose of phylogenetic studies of the *Cotesia* wasp species in the early 2000s by Kankare and colleagues [37, 42, 44, 45]. The DNA extracts have since been preserved in the freezer (-20 °C) at the University of Helsinki, Finland. The quality of each DNA extract was tested by PCR amplification of the mitochondrial cytochrome C oxidase subunit I gene (*COI* - primer pair LCO/HCO) [46]. The DNA extracts that did not amplify with the primers LCO/HCO after two PCRs were removed from further analyses.

To identify which potential symbionts could be found in this parasitoid wasp system, we first screened 56 *Cotesia* specimens from six species, and from four countries (Estonia, Finland, Spain, Sweden), for infection with five microbial symbionts (Table S1) known for manipulating other insect species’ reproductive systems. We screened for the bacteria *Spiroplasma* and *Cardinium* using the 16 S ribosomal RNA (*16 S r*RNA) gene [47–49], for the bacterium *Arsenophonus* by targeting the *23 S r*RNA gene [50], for *Wolbachia* using *Wolbachia*-specific primers amplifying the *wsp* (*Wolbachia surface protein*) gene and up to five conserved *Wolbachia* Multilocus Sequence Typing (MLST) markers: *coxA*, *fbpA*, *ftsZ*, *gatB* and *hcpA* [51], and for the fungal symbiont *Microsporidium* by amplifying the *18 S r*RNA gene [52]. We did not test for the presence of other microbial reproductive manipulators, such as *Rickettsia* [4]. We later screened all remaining *Cotesia* specimens for infection by the only detected symbiont: *Wolbachia*. One negative control (water sample) and one positive control were included in each PCR. Positive controls derived from DNA extractions of either a *Wolbachia*-infected *Ischnura elegans* specimen [53], an *Arsenophonus*-infected two-spot ladybird, *Spiroplasma*-infected *Drosophila* flies, *Cardinium*-infected midges (graciously provided by Prof. Hurst from Liverpool University), or a Microsporidium-infected *Melitaea* butterfly (Duplouy’s own lab). All primer sequences are given in Table S2. We Sanger sequenced the amplified genes on an ABI-3730 DNA Sequencer (Applied Biosystems) at the University of Helsinki, Finland, using only the forward primers for each gene. All *Wolbachia* MLST loci and *wsp* gene sequences were identified by comparing the resulting assemblies against the PubMLST database (https://pubmlst.org) with BLAST [54].

### Extracting, cleaning, and processing additional sequence material from NCBI repository

To put our findings on the most common symbiotic infection in *Cotesia* wasps in a larger phylogenetic context, we expanded the host species range and *Wolbachia* strain diversities of our study by screening for *Wolbachia* genomic material in the genomic data from *Cotesia* sequencing projects publicly available from NCBI Sequence Read Archive (SRA) database (https://www.ncbi.nlm.nih.gov/sra), and from the NCBI nucleotide database (https://www.ncbi.nlm.nih.gov/nucleotide). To do so, we first searched the SRA database using the keyword “*Cotesia*”, selecting “DNA” as source and “Genome” as strategy. With this approach, we identified 28 genome sequencing projects including both short-read and long-read data and representing six different *Cotesia* species (*C. congregata*, *C. flavipes*, *C. glomerata*, *C. rubecula*, *C. sesamiae*, and *C. vestalis* as synonym of *C. plutellae*) (Table S3).

We processed the short-read (Illumina) sequencing samples with Prinseq-lite (version 0.20.4) [55] to remove all sequences with at least one ambiguous nucleotide. The resulting reads were adapter trimmed and quality filtered using Trimmomatic (version 0.39) [56]. Quality assessment reports were obtained with FastQC [57] and summarized by MultiQC [58]. In contrast, the long-read sequences (Oxford Nanopore, ONT) were quality filtered using NanoFilt (version 2.7.1) [59], which excluded sequences with a mean base quality lower than ten and lengths lower than 1 kb. The quality of the processed specimens was evaluated with NanoStat (version 1.5.0) [59].

We then screened the Illumina samples for *Wolbachia* infection using Kraken2 (version 2.0.8) provided with a custom database of 142 *Wolbachia* publicly available reference genomes [60] (140 reference genomes from GenBank and two (*w*Di and *w*Ls) from http://nematodes.org/) (See Table S4). Samples with at least 1000 reads classified as *Wolbachia* according to Kraken2 [61] were then mapped against our *Wolbachia* reference genomes database using Bowtie2 (version 2.4.4) [62]. In contrast, the ONT sequencing data were directly aligned to *Wolbachia* reference genomes by Minimap2 (version 2.21) [63]. We used SAMtools (version 1.13) [64] to extract, merge, and sort reads properly mapped as pairs (mapping quality of 20 ) from the SAM file generated in the alignment step. For each alignment, the per-base read depth across two *Wolbachia* reference genomes (*w*MelPop strain GenBank CP046921.1 and *w*PipPel strain GenBank AM999887.1) was calculated using the SAMtools depth function and plotted in R with ggplot2 [65] (Fig. S2-S3). Mapped reads belonging to samples from the same BioSample were also processed as merged reads.

Finally, we built *Wolbachia* genome assemblies by individually assembling mapped reads from short- and long-read sequencing using the Unicycler pipeline (version 0.4.9) [66]. The quality and the completeness of the resulting genome assemblies were estimated by QUAST (version 5.0.2) [67] and BUSCO (version 5.4.3, Rickettsiales odb10 database) [68]. The assemblies, along with the two *Wolbachia* reference genomes mentioned above, were analysed using FastANI (version 1.3) [69]. FastANI estimates the Average Nucleotide Identity (ANI) metric, enabling the clustering of genomes from different individuals/organisms. This method facilitates the inference of the supergroup placement of *Wolbachia* strains by utilizing their entire genomes, and is a more comprehensive approach compared to using a limited set of markers. Annotation of *Wolbachia* assemblies and reference genomes was performed with Prokka (version 1.4.6) [70] using default settings. Subsequently, the protein sequences predicted by Prokka were uploaded into the OrthoVenn3 web server (https://orthovenn3.bioinfotoolkits.net); accessed date: 15 July 2023) for identification and comparison of orthologous clusters (Fig. S4). All final assemblies are available from Zenodo at 10.5281/zenodo.8422079.

### Identifying the CI-associated genes

To explore whether the *Wolbachia* strains analysed here may be causing CI in their hosts, we searched for the CI-associated genes, *cifA* and *cifB*, in the newly assembled *Wolbachia* genomes, using BLAST. For this purpose, we downloaded the amino acid sequences of CifA and CifB from various *Wolbachia* strains in the NCBI database (Organism: *Wolbachia*, Source: RefSeq only) using the keywords “cytoplasmic incompatibility CifA” or “cytoplasmic incompatibility CifB”. Subsequently, these amino acid sequences served as queries in two distinct searches: TBLASTN against *Wolbachia* assemblies and BLASTP against proteomes derived from the same assemblies. Homologs covering at least 70% of the length of the query, with an identity of at least 50%, and having an E-value cut-off of 10^− 10^ were aligned to the intact Cif homologs identified by Martinez et al. [71] using MUSCLE [72].

### Phylogenetic analyses

We inferred the phylogenetic relationships between the different *Wolbachia* strains of *Cotesia* wasps using the characterized *Wolbachia* MLST (*coxA*, *fbpA*, *ftsZ*, *gatB* and *hcpA*) and *wsp* genes sequences, from both DNA extracts and genomic sequence material from NCBI. To increase the list of *Wolbachia* strains included in our phylogeny, we screened the NCBI nucleotide database for any of the five *Wolbachia* MLST markers [51] and *wsp* gene [73] from any *Cotesia*, and some of their known Lepidoptera host species. As of the 10th January 2023, there was no record of *Wolbachia* strain from *Cotesia* species in the PubMLST database, however, we still recovered an additional 40 MLST and *wsp* sequences from four *Cotesia* species (*C. flavipes*, *C. glomerata*, *C. sesamiae* and *C. vestalis*), and four Lepidoptera host species (*Melitaea didyma*, *Chilo partellus*, *Pieris rapae*, *Plutella xylostella*) from NCBI. The sample size and geographic sampling locations are provided in Table S1 and illustrated by Figure S1. Additionally, we also recovered the sequences of 12 reference *Wolbachia* strains belonging to A-, B-, D- or F- supergroups and originating from different host species (https://pubmlst.org, *w*Au, *w*Bm, *w*Bol1, *w*Clec-F, *w*Ha, *w*Irr, *w*MelPop, *w*No, *w*Pel, *w*Ri, *w*Stri, *w*Vit), one strain from the butterfly *Danaus chrysippus (Nymphalidae)*, and three from the parasitoid wasp *Hyposoter horticola (Hymenoptera: Ichneumonidae)* (Table S1).

Individual MLST and *wsp* genes, and their concatenated alignments were produced using MAFFT [74], and manually curated in AliView [75]. We performed the phylogenetic analyses using RAxML [76] in raxmlGUI 2.0 [77] applying a general time reversible model with gamma-distributed rate variation across sites and a proportion of invariable sites (GAMMAGTR + I) on individual genes and concatenated alignments (Fig. S5 and S6). In all cases, node support was calculated by the rapid bootstrap feature of RAxML (100 replicates). The *Wolbachia* reference strains *w*Bm [78] and *w*Cle [79], which belong to the D- and F-supergroup, respectively, were used as outgroups to root the *Wolbachia* trees.

**Fig. 1:**
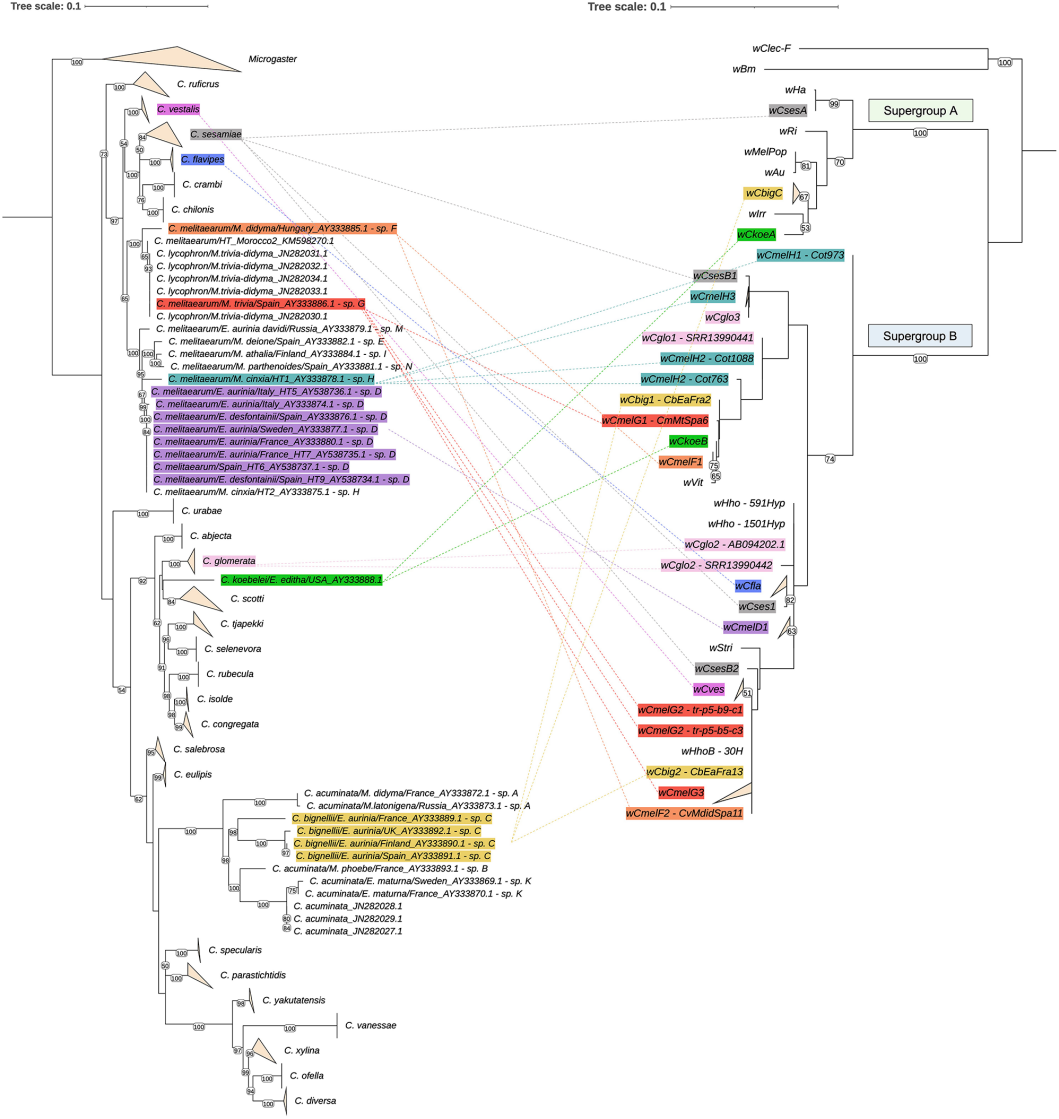
Comparison between *Cotesia* parasitoid lineages and *Wolbachia* strains from *Cotesia* species. The *Cotesia* maximum likelihood phylogenetic tree was inferred from the nucleotide sequence alignment (606 bp) of the mitochondrial *COI* gene. The *Wolbachia* maximum likelihood tree was based on concatenated alignment (2,559 bp) of the MLST and *wsp* markers and rooted using reference genomes from *Wolbachia* strains *w*Bm and *w*Clec belonging to the supergroups D and F, respectively. *Cotesia* species labelled A through N correspond to cryptic species described in [44]. The coloured lines link *Cotesia* host species to their respective *Wolbachia* strain infections; with a unique colour for each host species. Branches corresponding to different sequences obtained from different specimens within the same species, and sequences from different species but within the same genus (only in the case of the outgroup *Microgaster*), were collapsed and represented as orange triangles for visual clarity. Bootstrap support values > 50 are displayed at each node

To infer whether *Wolbachia* strain diversification is concordant with the *Cotesia* wasp species diversification, we inferred the phylogenetic relationships between *Cotesia* species using the *COI* sequences of *Cotesia* species, employing a maximum likelihood approach. We sampled all *COI* sequences deposited in GenBank for 39 different *Cotesia* species, including sequences from our *Cotesia* specimens previously deposited by [42, 44] (Table S5). As outgroups, we selected three species belonging to the *Microgaster* genus (Hymenoptera, Braconidae), namely *Microgaster nobilis*, *M. deductor* and *M. subcompletus* (see Table S5). We generated a *COI* sequence alignment of 606 bp with MAFFT, that we manually curated for misaligned regions using AliView. We constructed a maximum likelihood phylogeny from this alignment using IQTREE [80] under the best-fit model automatically selected by ModelFinder [81] (Fig. 1 and Fig. S7). Node support was estimated using ultrafast bootstrapping with 1,000 replicates [82].

The CifA and CifB proteins have previously been classified into at least five distinct phylogenetic clades (types I–V) with different degrees of compatibility [71, 83–85]. To determine the group to which the annotated Cif homologs from *Wolbachia* found in *Cotesia* hosts belong, we performed a phylogenetic analysis. The best-fit substitution model for the protein multiple sequence alignment was estimated using Modeltest-NG [86] in raxmlGUI 2.0 and based on the Akaike information criterion (AIC), it was determined to be a JTT + G4 + F model. A maximum likelihood phylogenetic tree was built using RAxML in raxmlGUI 2.0 software with 100 rapid bootstraps (Fig. S8). Tree visualization and figures were obtained with ITOL [87] using the bipartitions output trees produced by RAxML and the bootstrap consensus tree from IQTREE analysis.

## Results

### Detection of endosymbionts in *Cotesia* DNA extracts

Out of the 323 DNA extracts selected for *Wolbachia* screening, 282 were of good quality based on *COI* amplification, suggesting most of the specimens had been sufficiently preserved since extraction [42, 44].

The PCR amplifications for *Arsenophonus*, *Spiroplasma* or *Microsporidium* from 56 *Cotesia* specimens from four countries were negative (Table S1). There was one amplification using the *Cardinium 16S*rRNA primers in one unique specimen of *C. melitaearum* cryptic sp. H from Spain. However, our several attempts at sequencing this amplificon were not successful and hence we could also not confirm the presence of *Cardinium* in this *Cotesia* sample. In contrast, out of the 282 *Cotesia* samples of good quality, 50 (17.7%) carried the symbiotic bacterium *Wolbachia* (Tables 1, S1), representing at least eight *Cotesia* species parasitizing Melitaeini butterfly species (Fig. 1).

### Detection of endosymbionts in genome projects available in NCBI

By screening the 28 *Cotesia* genome projects (i.e. SRA projects) available on NCBI, we also identified 14 specimens (50%) containing at least 1000 reads classified as *Wolbachia* (Table S6). Ten specimens (six specimens from *C. glomerata*, one from *C. sesamiae* and three from *C. vestalis*) included *Wolbachia* reads distributed throughout the *Wolbachia* reference genomes (Figs. S2-S3), while the last four specimens only included reads with patchy coverage across the *Wolbachia* reference genomes. These last four projects were considered as potential false positive results for *Wolbachia* infection, with the *Wolbachia* reads representing potential contamination, or insertions of *Wolbachia* sequences in the *Cotesia* host genomes.

### *Wolbachia* strain diversity

Using the ten *Cotesia* genome projects found infected with *Wolbachia*, we partially assembled nine *Wolbachia* genomes. Three assemblies isolated from *C. glomerata*, exhibited BUSCO completeness of 86.8% (SRR13990441), 87.7% (SRR13990442), and 41.8% (SAMEA7283786) with corresponding total sizes of 1.10 Mbp, 1.08 Mbp, and 0.52 Mbp, respectively (See Tables S7-S8), while all other *Wolbachia* assemblies had a low number of BUSCO genes and were < 0.1 Mbp in size (Tables S7-S8). We were only able to extract between three and six MLST and *wsp* markers from the three largest *Wolbachia* assemblies.

Combining results obtained by direct amplification of the *Wolbachia* markers by PCRs and by screening the *Wolbachia* genomic assemblies built from *Cotesia* genomic sequences available on NCBI for those same markers, we obtained sequences from one to six markers for 38 (out of 61) *Wolbachia*-infected specimens (Table S1). We identified a total of 14 alleles for the *ftsZ* gene, nine for the *hcpA* gene, five for the *coxA* gene, six for *fbpA*, and six for *gatB* (See Table S1 for further details). This resulted in a concatenated alignment of 2559 bp, which allowed us to discriminate ten *Wolbachia* strains from ten *Cotesia* species (Table 1). We did not detect multiple infections in any of the individual *Cotesia* specimens, but two species carried several *Wolbachia* strains. Specimens of *C. koebelei* reared from *E. editha* from western North America carried either a supergroup A or a B *Wolbachia* strain, and Spanish specimens of *C. bignellii* carried a A-supergroup strain, while French specimens of the same species carried one of two B-supergroup strains (Fig. 1).

**Table 1:** Metadata for the *Cotesia* species and cryptic species found to be infected with *Wolbachia*: their butterfly host species, country of origin, and *Wolbachia* prevalence. Rows in grey highlight the specimens that were screened for all five symbionts (table S1), while rows in white include the specimens screened for *Wolbachia* only

Species	Host species (reared from)	Country	Infection rate (infected/total)	Strains detected
*C. acuminata* cryptic sp. B	*Melitaea phoebe*	Spain	24.4% (5/17)	Uncharacterized
*C. bignellii*	*Euphydryas aurinia*	France	100.0% (2/2)	*w*Cbig
*C. bignellii* cryptic sp. C	*Euphydryas aurinia*	Spain	50.0% (3/6)	*w*CbigC
*C. koebelei*	*Euphydryas editha*	USA	100.0% (2/2)	*w*CkoeA, *w*CkoeB
*C. melitaearum* cryptic sp. D	*Euphydryas aurinia*	Spain	10.8% (4/37)	*w*CmelD
*C. melitaearum* cryptic sp. F	*Melitaea didyma*	Spain	100% (12/12)	*w*CmelF
*C. melitaearum* cryptic sp. G	*Melitaea trivia*	Spain	92.9% (13/14)	*w*CmelG
*C. melitaearum* cryptic sp. H	*Melitaea cinxia*	Finland	11.1% (6/54)	*w*CmelH1
*C. melitaearum* cryptic sp. H	*Melitaea cinxia*	Russia	27.3% (3/11)	Uncharacterized
*C. glomerata*	*Pieris sp.*		75% (6/8)	*w*Cglo
*C. sesamiae*	*Stem boring moths*		100% (1/1)	Uncharacterized
*C. vestalis*	*Plutella sp.*		75% (3/4)	Uncharacterized

### Analyses of the *Wolbachia* genomic assemblies

By comparing the predicted proteomes of our two largest *Wolbachia* assemblies with an > 50% BUSCO completeness against those of the two *Wolbachia* reference genomes (*w*MelPop and *w*PipPel) using Prokka, we identified 954 protein-coding genes, 30 tRNAs, and one rRNA in the SRR13990441 assembly, and 996 protein-coding genes, 32 tRNAs, and three rRNAs in the SRR13990442 assembly (Table S9). In contrast, the two reference genomes, *w*MelPop and *w*PipPel, contained 1304 and 1410 protein-coding genes, 34 tRNAs, and three rRNAs, respectively (Table S9). The comparison using the Orthovenn 3 web server showed a total of 1057 conserved orthologs in all four strains, with 590 of these being single copy. All four strains shared 639 ortholog clusters (Fig. S4). The SRR13990442 assembly contains 876 orthologs, while SRR13990441 has 875, and they both share 71 unique orthologs with the reference B-supergroup *Wolbachia w*PipPel, but only 21 with the A-supergroup *Wolbachia w*MelPop reference (Fig. S4). Similarly, the ANI analysis, which calculates the average nucleotide identity among orthologous gene pairs shared between two genomes, revealed a high similarity between *w*PipPel, SRR13990441, and SRR13990442, with ANI values around 98% in pairwise comparisons (Table S10). In contrast, *w*MelPop displayed a lower ANI (~ 85%) in pairwise comparisons with *w*PipPel, SRR13990441, and SRR13990442 (Table S10). Altogether, these results suggest the two *Wolbachia* assemblies from *Cotesia* belong to the B-supergroup *Wolbachia*.

Finally, we partially extracted the CI-associated genes from our *Wolbachia* assemblies. With this, we identified one copy of a Type I CifA in the SRR13990441 assembly (Table S11, Fig. S8), and a truncated/partial copy of *cifB* in both the SRR13990441 assembly (contig 109, position 1492–3201) and the SRR13990442 assembly (contig 221, position 1-1624). The sequences of the *cif*B gene from our *Wolbachia* assemblies were highly similar to that previously characterized from the fig wasp *Kradibia gibbosae* (Hymenoptera: Chalcidoidea) (WP_275944372.1), without any report of the role played by the symbiont in this host species [88].

### Phylogenetic analyses

Our phylogenetic tree of the *COI* mitochondrial gene of 39 *Cotesia* species shows that the *Cotesia* wasps parasitizing Melitaeini butterflies belong to three distinct clades (See Fig. 1, S5-6:

- Clade 1 includes *C. melitaearum* cryptic species (D, E, F, G, H, I, M, N),
- Clade 2 includes *C. koebelei*,
- Clade 3 includes *C. bignellii* cryptic species C, and *C. acuminata* cryptic species (A, B, and K).

The *Wolbachia* phylogeny confirms that all *Wolbachia* strains characterized from *Cotesia* belonged to the A- and B-supergroups, with the majority (49/53, 92.4%) belonging to the B-supergroup (Fig. 1). Despite fewer representative taxa per phylogeny and lower resolution, phylogenies based only on individual gene alignments maintained similar sample groupings, with conserved strain assignment to supergroups A and B (Fig. S5), thus suggesting no recombination has occurred between the strains of the two supergroups in these *Cotesia* species. A visual comparison supports the lack of congruence and co-phylogeny between the maximum likelihood trees of *Cotesia* and their symbiotic strains. Phylogenetically close *Wolbachia* strains were found in phylogenetically distant *Cotesia* host species (Fig. 1, & S5-6), or in both a butterfly host and their *Cotesia* parasitoid (i.e. the *w*Mdid from a *M. didyma* butterfly and the *w*CmelF strain from *Cotesia* wasps emerging from Spanish *M. didyma* butterflies, Fig. S5).

## Discussion

We built up from early studies to bring some light on the possible role(s) of endosymbionts on the evolutionary history of *Cotesia* parasitoid wasps, particularly on the divergence between sympatric cryptic species. The complex of *Cotesia* wasps parasitizing Melitaeini butterflies belongs to three distinct clades, as previously shown by [44]. These three *Cotesia* clades are mostly specialists to the Melitaeini butterflies, but clustering based on the Lepidoptera host is not conserved across the genus *Cotesia*. Indeed, closely related *Cotesia* species to each of the three clades have been described as parasitoid wasps of divergent Lepidoptera. For example, *C. glomerata* parasitizes *Pieris sp*. butterflies (Pieridae), while *C. specularis* emerges from *Lampides boeticus* (Lycaenidae), and other *Cotesia* species are known to attack diverse moths (i.e. *Chilo sp*. for *C. flavipes*, or *Plutella sp*. for *C. vestalis*), with each of these Lepidoptera species feeding on a wide diversity of host plants. In their early studies, Kankare et al. [37, 42] suggested that direct competition between *Cotesia* wasps for the Melitaeini butterfly host species might have driven the divergence between their parasitoid wasp species and cryptic species. We did not detect the symbionts *Arsenophonus*,* Cardinium*,* Microsporidium* and *Spiroplasma*, and did not test for infection with *Rickettsia*, however the endosymbiotic bacterium *Wolbachia* was found in 61 (17.9%) of all our samples, covering 11 *Cotesia* species (52.4%) out of 21 included in the study. As in previous studies on *Cotesia* wasps species [39–41], and in insects in general [89, 90], such *Wolbachia* prevalence is still likely an underestimate of the true infection prevalence in the entire *Cotesia* genus. This is because our study covers only a small number of *Cotesia* species, populations and individuals representing only part of their geographic distributions [33].

Although the commonly used MLST markers have been criticised for being too conserved to allow reliable strain differentiation or infer precise phylogenetic relationships of closely related *Wolbachia* strains [91], comparison of the phylogenetic trees from *Cotesia* hosts and their *Wolbachia* symbionts clearly showed that distantly related *Cotesia* species share similar *Wolbachia* strains. Such lack of concordance between the host and the symbiont phylogenies has been previously described in diverse systems [22, 29]. This pattern suggests that *Wolbachia* strains have transferred horizontally, and not strictly vertically between mothers and their offspring. Because many *Cotesia* species occur in sympatry, sharing either their geographical ranges, their local habitats, their hosts, which in some cases also share the same host plants [37, 92, 93], the *Cotesia* species complex offers plausible opportunities for *Wolbachia* to transfer horizontally:

First, divergent *Cotesia* wasps might have acquired their *Wolbachia* infections while parasitising infected caterpillars. Between others, studies by Vavre et al. [29], and Qi et al. [94] provide evidence of such horizontal transfers between *Drosophila* flies and their parasitoid wasps, and between whiteflies and their parasitoid wapss, respectively. Although *Wolbachia* was previously detected in *M. didyma* [95, 96], *M. athalia*, *M. britomartis* [97], *M. phoebe*, *M. ornata* [98] and *M. cinxia* [22], genetic sequences for most of those strains were not publicly available (Nov. 2023). However, we did find that a sequence from the *wsp* gene of the *Wolbachia* strain infecting *C. melitaearum*, parasitizing *M. didyma* caterpillars, was very similar to the *wsp* sequence from a strain infecting *M. didyma*. This suggests that this *Wolbachia* strain might have transferred between the *Cotesia* wasp and the Lepidoptera host, as also shown in *Nasonia* wasps and their *Drosophila* hosts [30]. But this is not always the case, as other strains characterized from other Lepidoptera species (i.e. *w*Cpar from *Chilo partellus*, *w*Prap from *Pieris rapae*, and *w*Pxyl from *Plutella xylostella*) were phylogenetically divergent from the strains found in the *Cotesia* wasps infecting those Lepidoptera (*C. flavipes*, *C. glomerata*, *C. vestalis*, respectively).

Second, *Wolbachia* could be exchanged between parasitoid wasps simultaneously parasitising the same host caterpillar. Such hypothesis was previously tested by [29], who found that *Leptopilina*, *Trichopria* and *Asobara* parasitoids of *Drosophila* flies can share identical *Wolbachia* bacteria, at least based on their *wsp* gene sequences. In Åland, *M. cinxia* is commonly parasitized by several parasitoid wasps [99]. Out of these, *Hyposoter horticola* is known to carry *Wolbachia* [100], and we showed that this *Wolbachia* strain (*w*Hho) is phylogenetically closely related to the *Wolbachia* characterized from *C. melitaearum*. These results suggest that at least some *Wolbachia* might also transfer horizontally between divergent parasitoid species sharing the same Lepidoptera hosts, or between the parasitoid wasps and their Lepidoptera hosts. In the future, hypotheses presented above could be more comprehensively tested in the *Cotesia* system by simultaneously screening *Cotesia* wasps and their Lepidoptera hosts for endosymbiotic infections.

The lack of co-divergence between the *Wolbachia* strains and their associated *Cotesia* lineages does not allow us to fully reject the hypothesis that the symbiont might be involved in the restricted gene flow between at least some of the sympatric *Cotesia* lineages [101]. For example, CI could occur between *Cotesia* lineages that carry divergent *Wolbachia* strains, such as *C. melitaearum* sp. F and G, or between *Cotesia* lineages of different infection status, such as *C. melitaearum* sp. D and E. In the future, experimental rearing [102, 103] and crossing between lineages, associated with microscopy imagery, should confirm the expression of CI between different host lineages. Indeed, because CI causes visible morphological abnormalities of sperm in the testes of infected males [104, 105], or cytological embryonic defects, microscopic approaches may be used to confirm post-mating isolation between *Cotesia* lineages, as shown previously in *Culex pipiens*,* Drosophila simulans* and the parasitoid wasp, *Nasonia* [106–109]. Here, we isolated a complete homolog of the *cifA* gene and a partial homolog of *cifB* gene, which code for the *Wolbachia*-induced CI phenotype in other species [83, 110, 111]. However, with the growing general interest for whole genome sequencing of parasitoid wasps, especially of species used as agricultural pest control agents, we expect more *Cotesia* genomic projects to be completed in the near future. These projects will hopefully assemble and analyse the whole genomes of *Wolbachia* and other endosymbiotic species associated with *Cotesia* hosts, and soon provide material for holistic estimate of the endosymbionts patterns of diversity, and their function(s) in this complex of parasitoid wasp species.

### Electronic supplementary material

Below is the link to the electronic supplementary material.

Supplementary Material 1

Supplementary Material 2

Supplementary Material 3

Supplementary Material 4

Supplementary Material 5

Supplementary Material 6

Supplementary Material 7

Supplementary Material 8

Supplementary Material 9

Supplementary Material 10

Supplementary Material 11

Supplementary Material 12

Supplementary Material 13

Supplementary Material 14

Supplementary Material 15

Supplementary Material 16

Supplementary Material 17

Supplementary Material 18

Supplementary Material 19

Supplementary Material 20

Supplementary Material 21

## Data Availability

All sequence alignments and final assemblies are available from Zenodo at 10.5281/zenodo.8422079. All sequences were deposited on NCBI with the accession numbers OR597552-OR597565 (wsp), OR608488 - OR608500 (hcpA), OR640995-OR641006 (coxA), OR641007-OR641026 (ftsZ), OR641031- OR641044 (gatB), and OR641048-OR641057 (fbpA).

## Abbreviations

CI: Cytoplasmic Incompatibility
MLST: Multi Locus Sequence Typing
