# Supplementary figures and images for: Wolbachia strain diversity in a complex group of sympatric cryptic parasitoid wasp species
Source: BMC Microbiol. 2024 Sep 2;24:319. doi: 10.1186/s12866-024-03470-7 (PMC11368008; doi:10.1186/s12866-024-03470-7)

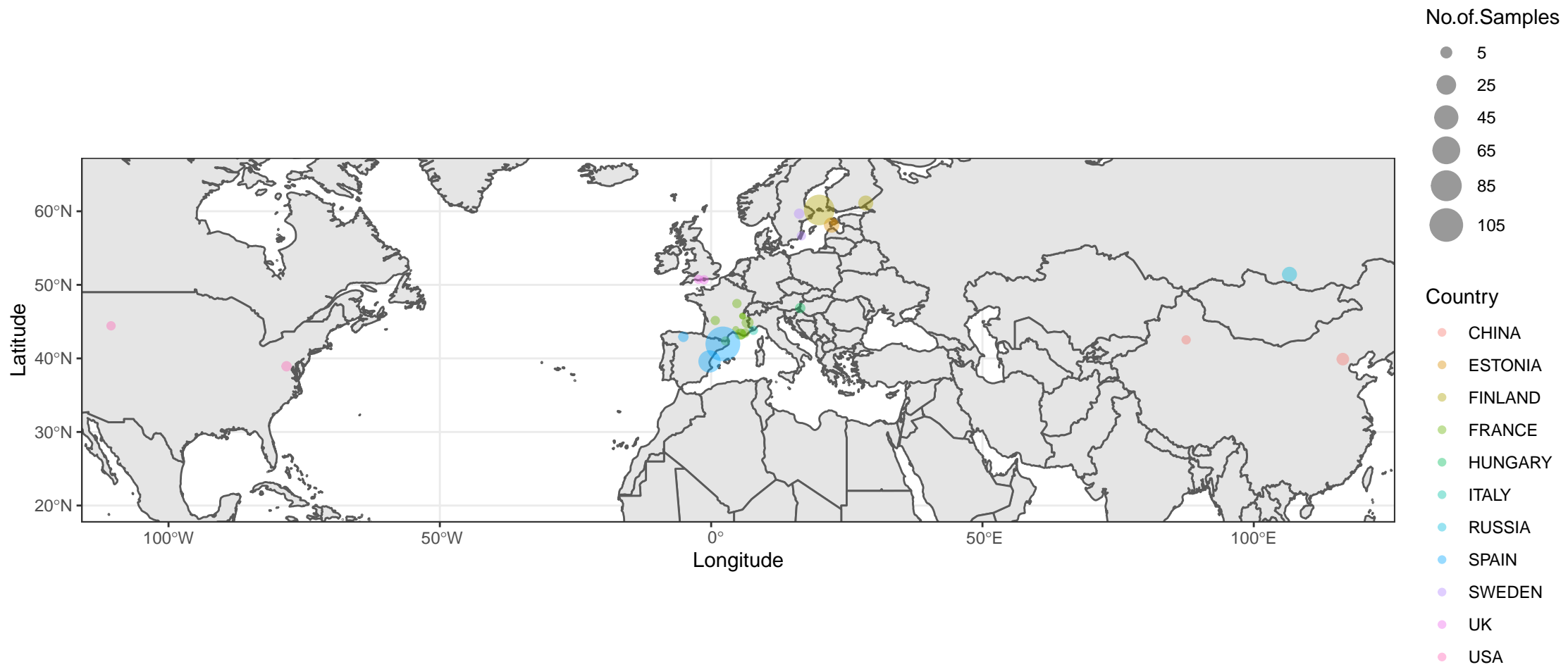

Supplement: Supplementary file 1 — Supplementary Material 1 [file 12866_2024_3470_MOESM1_ESM.pdf]

# Coverage across wPipPel genome

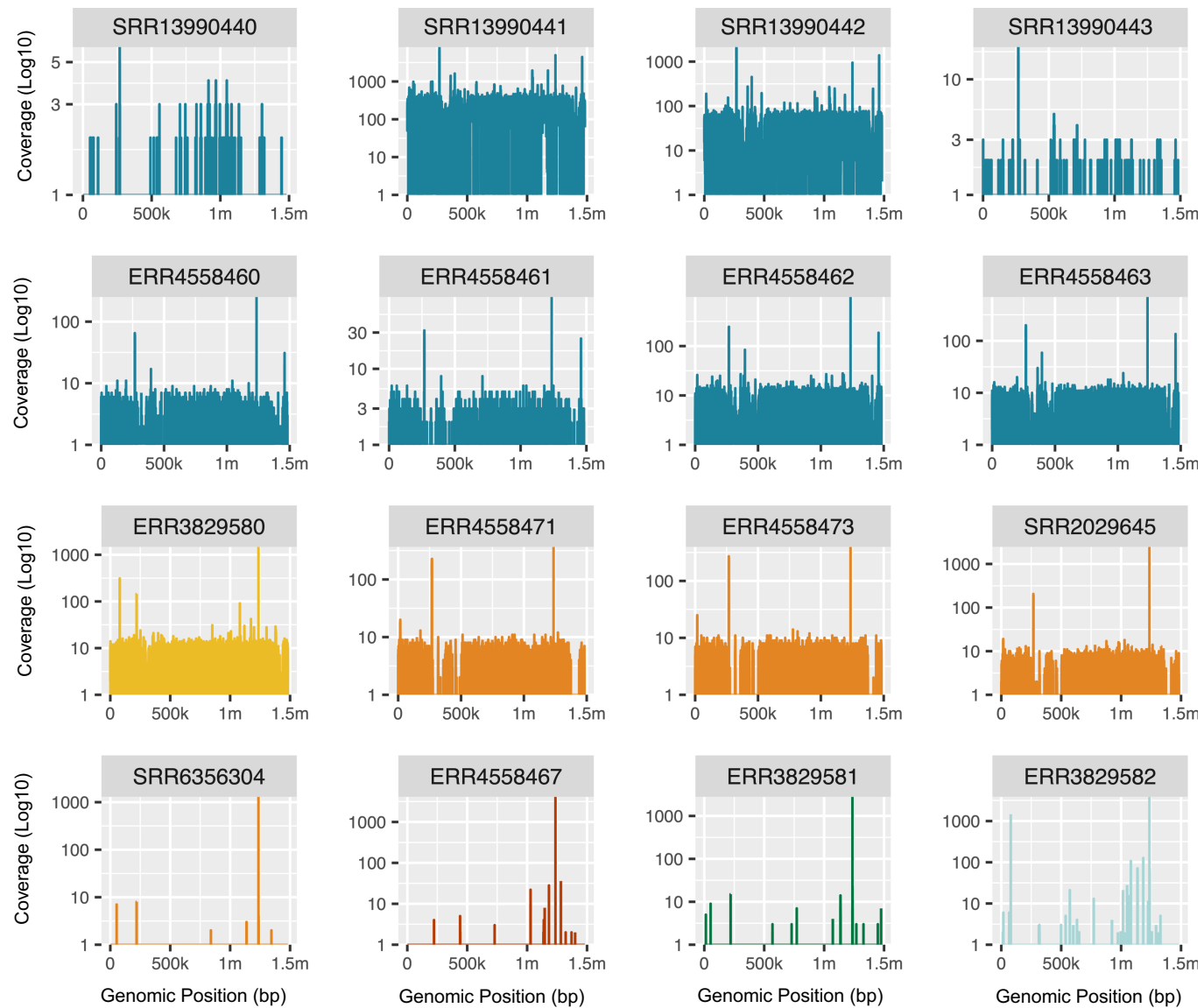

Supplement: Supplementary file 3 — Supplementary Material 3 [file 12866_2024_3470_MOESM3_ESM.pdf]

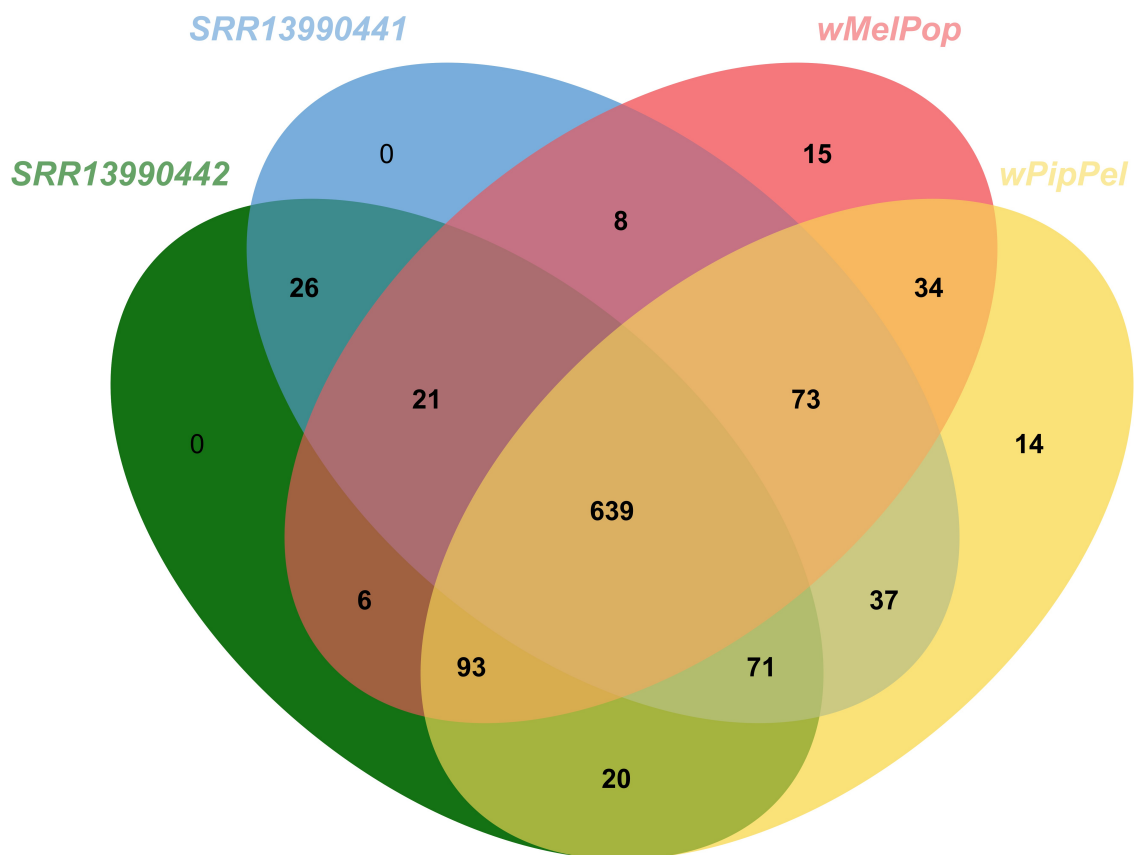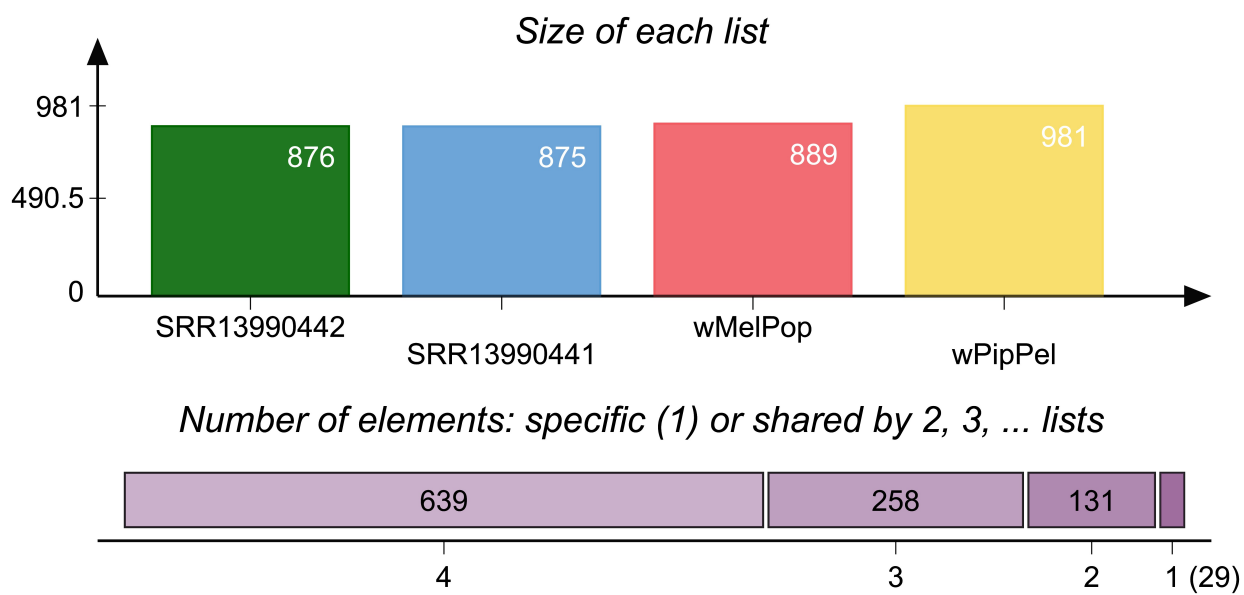

Supplement: Supplementary file 4 — Supplementary Material 4 [file 12866_2024_3470_MOESM4_ESM.pdf]

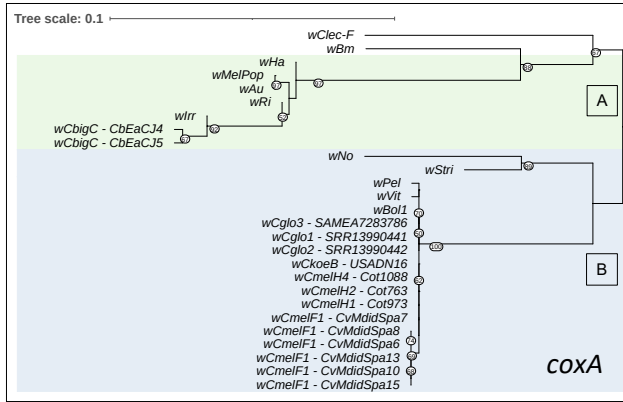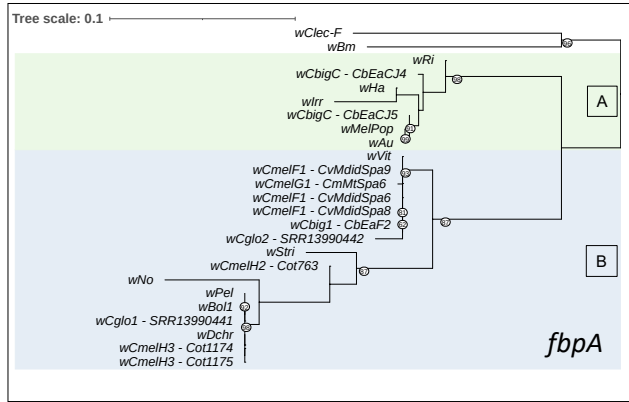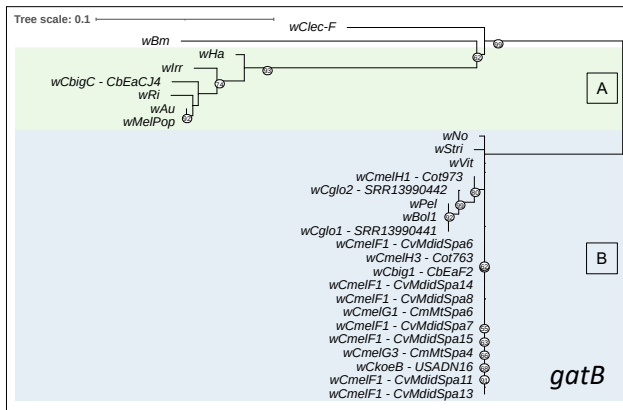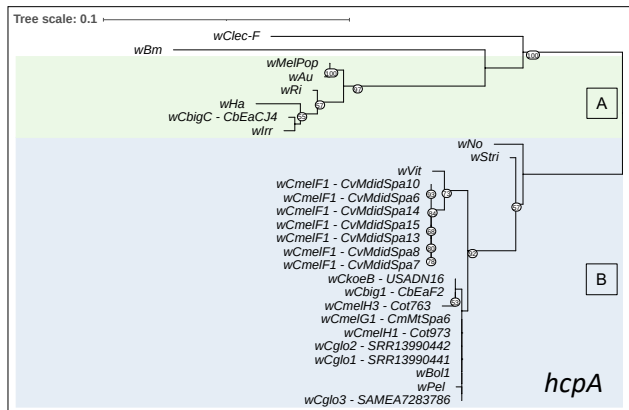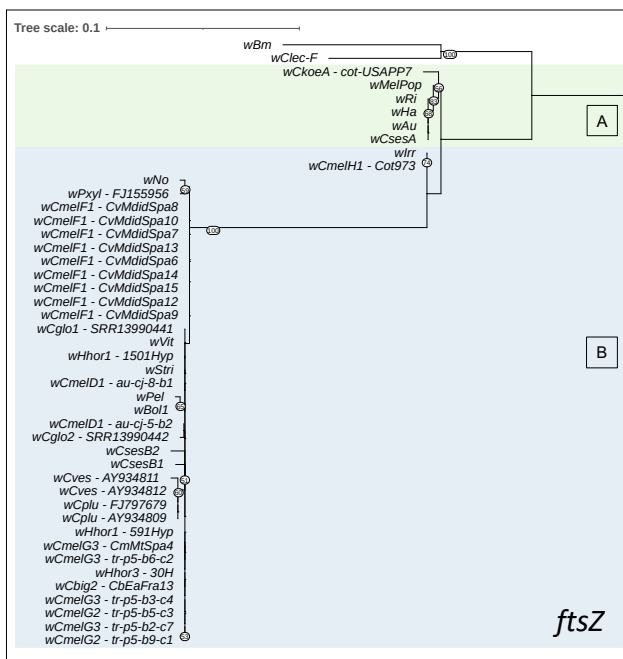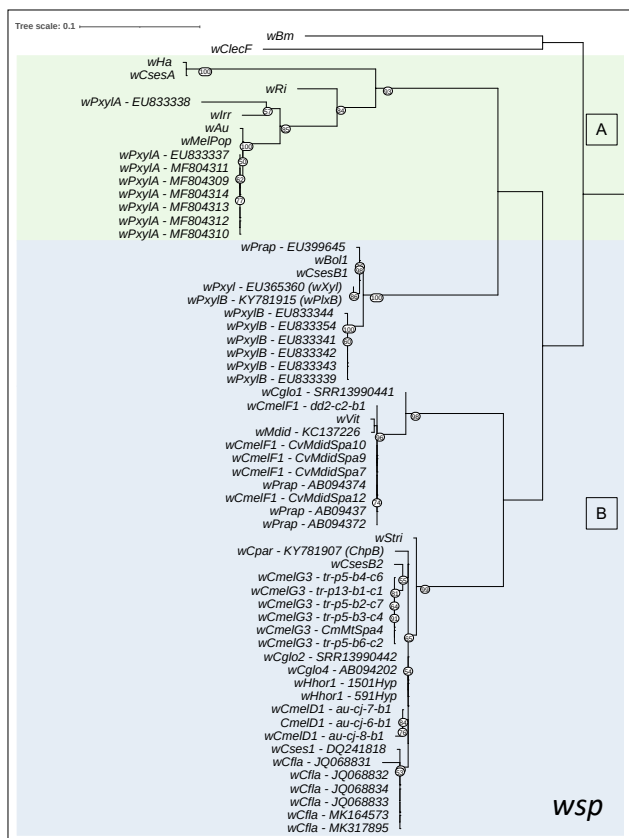

Supplement: Supplementary file 5 — Supplementary Material 5 [file 12866_2024_3470_MOESM5_ESM.pdf]

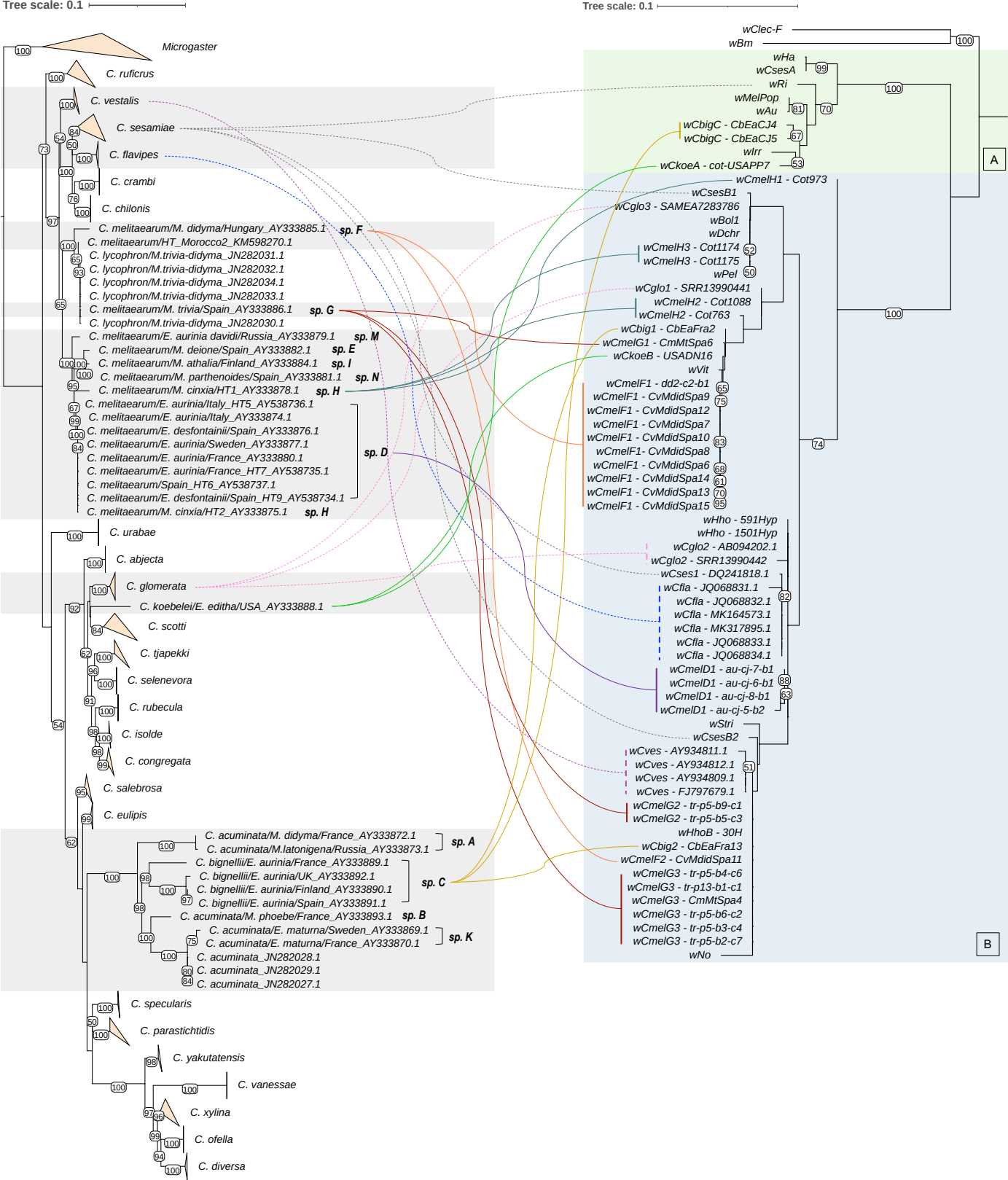

Supplement: Supplementary file 6 — Supplementary Material 6 [file 12866_2024_3470_MOESM6_ESM.pdf]

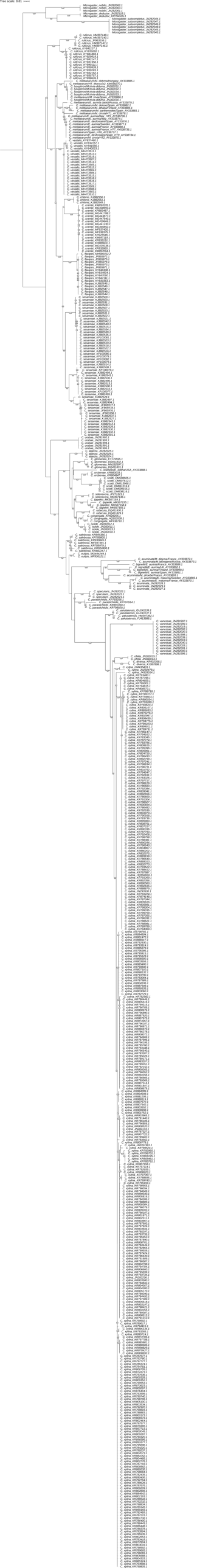

Supplement: Supplementary file 7 — Supplementary Material 7 [file 12866_2024_3470_MOESM7_ESM.pdf]

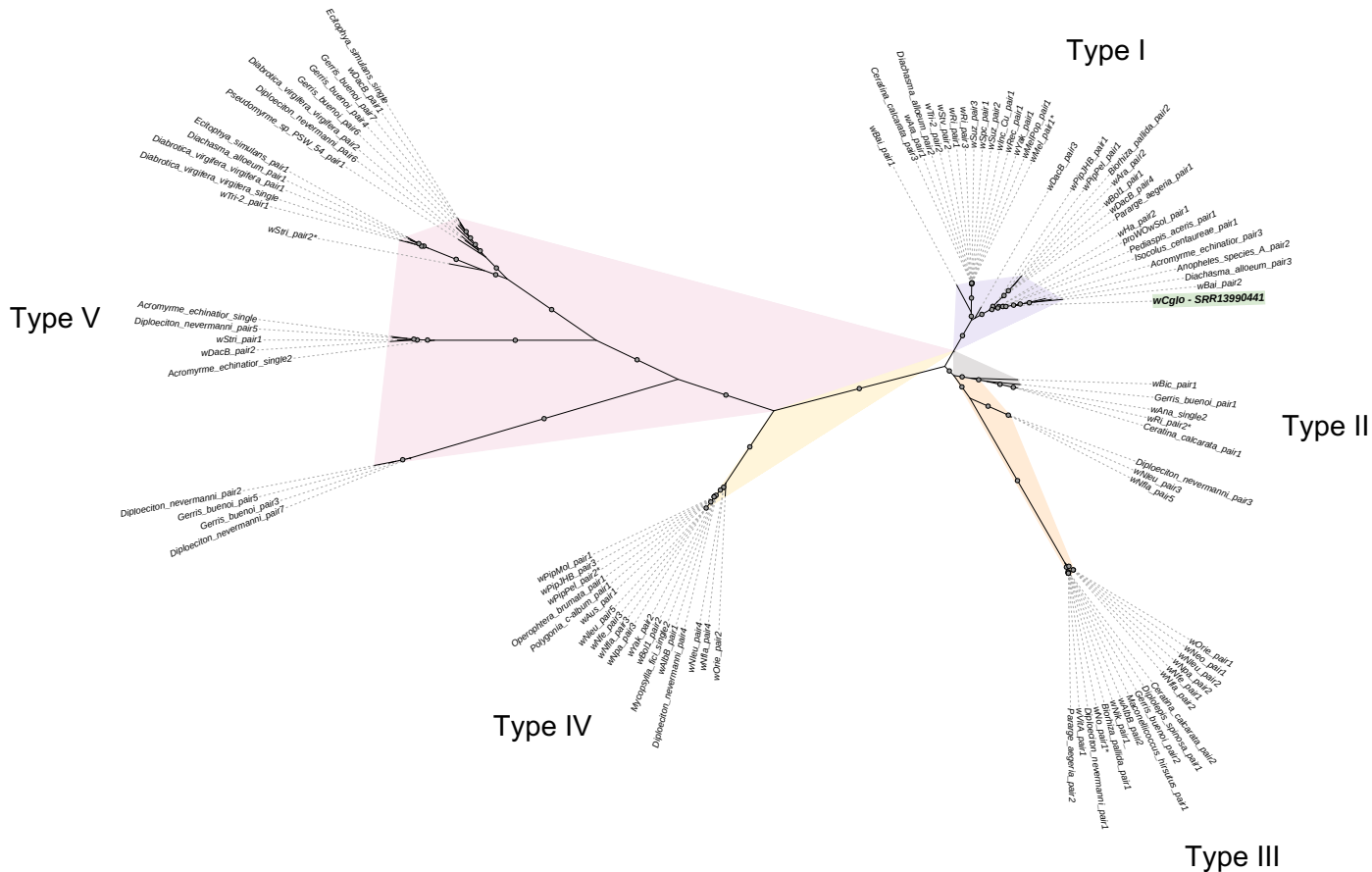

Supplement: Supplementary file 12 — Supplementary Material 12 [file 12866_2024_3470_MOESM12_ESM.pdf]
