# Supplementary material for: Wolbachia strain diversity in a complex group of sympatric cryptic parasitoid wasp species
Source: BMC Microbiol. 2024 Sep 2;24:319. doi: 10.1186/s12866-024-03470-7 (PMC11368008; doi:10.1186/s12866-024-03470-7)
Supplement: Supplementary file 2 — Supplementary Material 2 [file 12866_2024_3470_MOESM2_ESM.pdf]

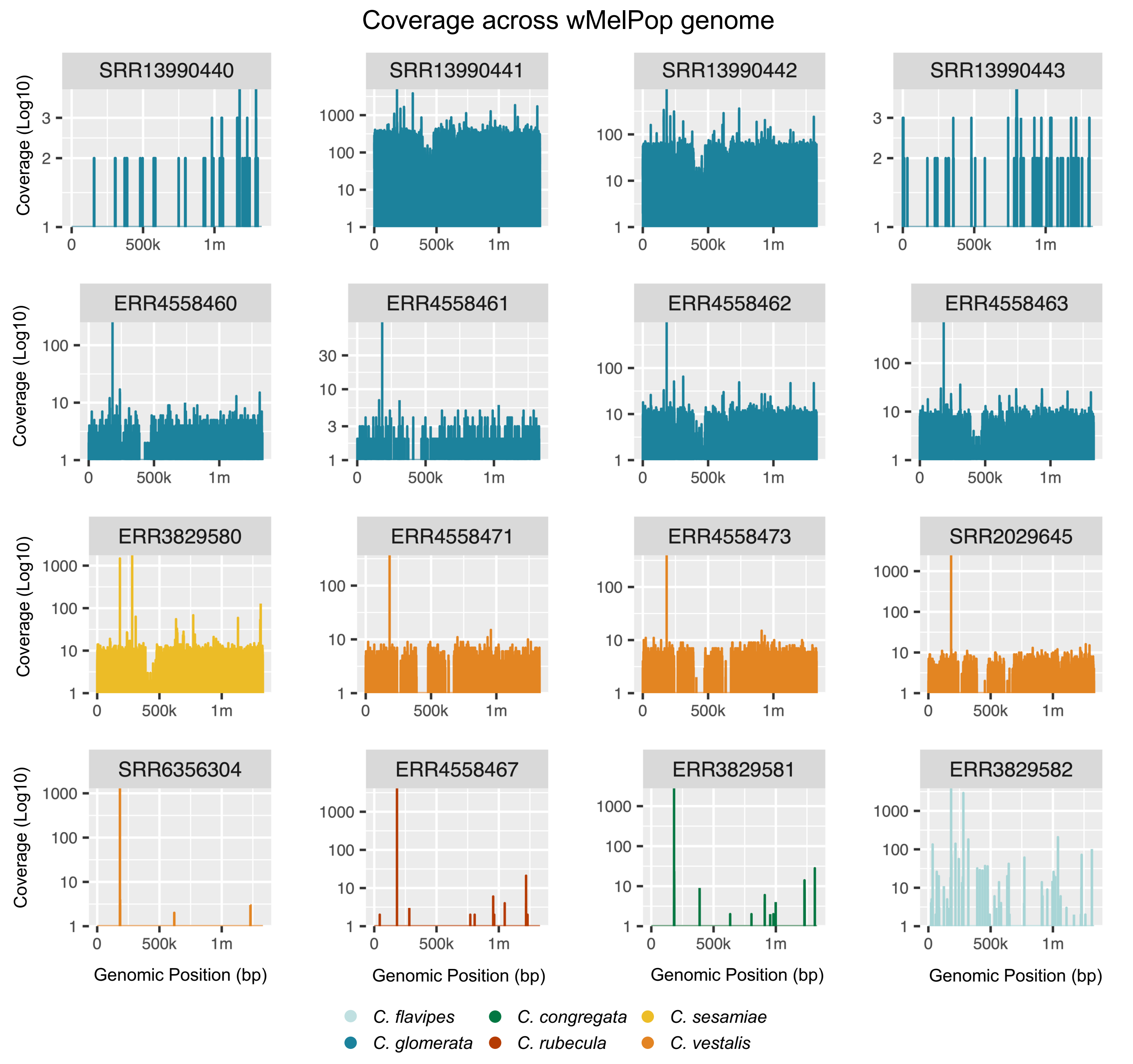

Coverage across wMelPop genome
SRR13990440
SRR13990441
SRR13990442
SRR13990443
ERR4558460
ERR4558461
ERR4558462
ERR4558463
ERR3829580
ERR4558471
ERR4558473
SRR2029645
SRR6356304
ERR4558467
ERR3829581
ERR3829582
Coverage (Log10)
Genomic Position (bp)
0
500k
1m
C. flavipes
C. glomerata
C. congregata
C. rubecula
C. sesamiae
C. vestalis
