# Supplementary material for: Wolbachia strain diversity in a complex group of sympatric cryptic parasitoid wasp species
Source: BMC Microbiol. 2024 Sep 2;24:319. doi: 10.1186/s12866-024-03470-7 (PMC11368008; doi:10.1186/s12866-024-03470-7)
Supplement: Supplementary file 21 — Supplementary Material 21 [file 12866_2024_3470_MOESM21_ESM.docx]

**Supplementary data**

**Table S1 – Metadata for all *Cotesia* specimens included in the study**: IDs, species name, host species, country of origin, PCR and sequencing results. Rows in grey highlight the specimens that were screened for all five symbionts, while rows in white only include the specimens screened for *Wolbachia* only. MD= Missing data not provided by collector, NA= Non applicable if not tested or failed.

**Table S2 – Primer pairs used in the study:** the expected amplified fragment size, the respective sequences and reference**.**

**Table S3 – Metadata for all the samples retrieved from NCBI:** The first sheet contains information about DNA sequencing of *Cotesia* samples from NCBI SRA (e.g., sample ID, assay type, BioProject, BioSample, species name). The second sheet contains information about gene sequences of *Wolbachia* infecting *Cotesia* species from NCBI Nucleotide (i.e., GenBank ID, description, host species).

**Table S4 – *Wolbachia* reference genomes included in the reference genome database.** The 'X' symbol marks missing data, as the strain *w*Dim and *w*Ls were not collected from the NCBI database.

**Table S5 – Metadata for COI genes from NCBI Nucleotide:** GenBank ID, description, host species.

**Table S6 – Summary of the SRA samples with hits to *Wolbachia* based on Kraken2:** the sample ID, species name, BioSample identifier, and the number of *Wolbachia* reads. Rows in green highlight the specimens with at least 1000 reads classified as *Wolbachia***.**

**Table S7 – Genome assembly completeness according to BUSCO.**

**Table S8 – Genome assembly metrics calculated by QUAST.**

**Table S9 – Statistics relating to the annotated features found by Prokka.**

**Table S10 – Pairwise Average Nucleotide Identity (ANI) values between *Wolbachia* reference genomes (wMelPop and wPipPel) and the strains isolated from *C. glomerata* (SRR13990441 and SRR13990442) calculated using FastANI.**

**Table S11 - BLAST Search of CI Genes.** Output of BLASTP and TBLASTN analyses using a database of CifA and CifB from different *Wolbachia* strains as query against *Wolbachia* assemblies and proteomes from *C. glomerata*.

**Table S12 – MLST profiles assigned by the *Wolbachia* PubMLST database (**[**https://pubmlst.org/wolbachia/**](https://pubmlst.org/wolbachia)**).**

**Figure S1 – Map depicting the geographical distribution of the sampling sites by country.**

**Figure S2 – Coverage plots illustrating the distribution of *Wolbachia* reads identified in publicly available *Cotesia* sequencing projects across the wMelPop reference genome**. The coverage was calculated using samtools depth and is displayed at every position on the reference genome.

**Figure S3 – Coverage plots illustrating the distribution of *Wolbachia* reads identified in publicly available *Cotesia* sequencing projects across the wPipPel reference genome.** The coverage was calculated using samtools depth and is displayed at every position on the reference genome.

**Figure S4 – Venn diagram generated using OrthoVenn3 online service showing the distribution of protein clusters shared and unique among the reference genomes *w*MelPop and *w*PipPel, and the *Wolbachia* strains isolated from *C. glomerata* SRR13990441 and SRR13990442.**

**Figure S5 – Maximum likelihood phylogenetic trees** **inferred from individual *Wolbachia* MLST and *wsp* genes using GAMMAGTR+I substitution model.** Bootstrap support values > 50 are depicted at each node as grey circles.

**Figure S6 – Comparison between *Cotesia* parasitoid lineages against the *Wolbachia* strains from *Cotesia* species (extended version of Figure 1).** The *Cotesia* maximum likelihood phylogenetic tree was inferred from the nucleotide sequence alignment (606 bp) of the mitochondrial COI gene. The *Wolbachia* maximum likelihood tree was based on concatenated alignment (2,559 bp) of the MLST and *wsp* markers and rooted using reference genomes from Wolbachia strains *w*Bm and *w*Clec belonging to the D- and F-supergroups, respectively. The coloured lines link *Cotesia* host species to their respective *Wolbachia* strain infections; with a unique colour for each host species. Solid lines represent *Cotesia* species that parasitize Melitaeini butterflies, whereas dashed lines are used for *Cotesia* species that parasitize other Lepidopteran species. Branches corresponding to different sequences obtained from different specimens within the same species, and sequences from different species but within the same genus (only in the case of the outgroup *Microgaster*), were collapsed and visually represented as orange triangles for visual clarity. Bootstrap support values > 50 are displayed at each node.

**Figure S7 – Maximum likelihood phylogeny of the genus *Cotesia* inferred from the nucleotide sequence alignment (606 bp) of the mitochondrial COI gene.** Bootstrap support values > 50 are depicted at each node as grey circles.

**Figure S8 – Maximum likelihood phylogeny based on the alignment of CifA amino acid sequences using JTT+G4+F substitution model**. Previously defined CifA Types (I-V) are labelled and colour-shaded. Bootstrap support values > 50 are depicted at each node as grey circles.
